# Neuregulin-1 signaling regulates cytokines and chemokines expression and secretion in granulosa cell

**DOI:** 10.1186/s13048-022-01021-0

**Published:** 2022-07-26

**Authors:** Saswati Banerjee, Sameer Mishra, Wei Xu, Winston E. Thompson, Indrajit Chowdhury

**Affiliations:** 1 Department of Physiology, Morehouse School of Medicine, Atlanta, GA USA; 2 Department of Obstetrics and Gynecology, Morehouse School of Medicine, 720 Westview Drive Southwest, Atlanta, GA 30310 USA

**Keywords:** Neuregulin-1, Granulosa cell, Cytokines, Chemokines

## Abstract

**Background:**

Granulosa cells (GCs) are multilayered somatic cells within the follicle that provide physical support and microenvironment for the developing oocyte. In recent years, the role of Neuregulin-1 (NRG1), a member of the EGF-like factor family, has received considerable attention due to its neurodevelopmental and cardiac function. However, the exact physiological role of NRG1 in GC is mainly unknown. In order to confirm that NRG1 plays a regulatory role in rat GC functions, endogenous NRG1-knockdown studies were carried out in GCs using RNA interference methodology.

**Results:**

Knockdown of NRG1 in GCs resulted in the enhanced expression and secretion of the cytokines and chemokines. In addition, the phosphorylation of PI3K/Akt/ERK1/2 was significantly low in GCs under these experimental conditions. Moreover, in vitro experimental studies suggest that tumor necrosis factor-α (TNFα) treatment causes the physical destruction of GCs by activating caspase-3/7 activity. In contrast, exogenous NRG1 co-treatment of GCs delayed the onset of TNFα-induced apoptosis and inhibited the activation of caspase-3/7 activity. Furthermore, current experimental studies suggest that gonadotropins promote differential expression of NRG1 and ErbB3 receptors in GCs of the antral follicle. Interestingly, NRG1 and ErbB3 were intensely co-localized in the mural and cumulus GCs and cumulus-oocyte complex of pre-ovulatory follicles in the estrus stage.

**Conclusions:**

The present studies suggest that gonadotropins-dependent NRG1-signaling in GCs may require the balance of the cytokines and chemokines expression and secretion, ultimately which may be supporting the follicular maturation and oocyte competence for ovulation and preventing follicular atresia.

**Supplementary Information:**

The online version contains supplementary material available at 10.1186/s13048-022-01021-0.

## Background

The gonadotropins play a central role in promoting a cascade of events in ovarian antral and preovulatory (PO) follicles (F) that are essential for the ovulation of a fertilizable oocyte [1]. Gonadotropins-dependent granulosa cells (GCs) provide important metabolites and steroid hormones and cooperate with resident and infiltrating immune cells to produce multiple autocrine and paracrine factors for follicular growth maturation and ovulation and support corpus luteum (CL) formation [2–5]. The GCs are multilayered somatic cells within the follicle and create a unique microenvironment by maintaining a close anatomical relationship for oocyte development [2–7]. The formation and maturation of antral and PO-follicle involves an autocrine-paracrine dialogue between the oocyte and GC-layers along with theca cell layers, principally mediated by an extensive array of factors including epidermal growth factor (EGF)-like peptides, steroid hormones (mainly estrogen, E2 and progesterone, P4), and locally produce cytokines and chemokines [1, 3, 8–10].

Cytokines are small, soluble, and diverse pleiotropic immunoregulatory signaling proteins with a short half-life, whereas chemokines are a group of secreted proteins within the cytokine family [11]. Cytokines and chemokines create a chemotactic immune and non-immune gradient in the follicle [2–4, 12, 13]. In turn, these cytokines and chemokines govern locally follicular cell fate and homeostasis by regulating cell survival, death, proliferation, and differentiation [3, 10, 11, 13, 14]. Given these pivotal roles and their ease of detection in follicular fluid, cytokines and chemokines have been considered attractive biomarkers of oocyte maturational status and successfully assisted reproductive outcomes [5, 10]. Despite this evidence, our understanding of physiological regulations and interactions of these cytokines during follicular development and maturation remains incomplete and is still limited to overly simplistic descriptions of their interrelationships [2–5, 10, 15–18].

Gonadotropins-dependent transactivation of the EGF-family members is indispensable for the cumulus (C) and mural (M) -GCs (CGC, MGC) function to support oocyte reentry into the meiotic cell cycle to synthesize the extracellular matrix surrounding the oocyte that causes cumulus expansion and for follicle rupture [3, 19–23]. EGF-like growth factors are synthesized as integral membrane precursors and shed from the cell surface by proteolytic cleavage of the ectodomain and are responsible for the biological activities [24, 25]. In recent years, an important concept has emerged that neuregulin-1 (NRG1), a member of the EGF-like factor family, is gonadotropin-dependent differentially expressed in GCs and supports cell survival, P4 synthesis, and oocyte maturation [21–23]. ErbB3 and ErbB4 are two bona fide receptors for NRG1 [26]. Upon NRG1 binding, ErbB3 forms a heterodimer with ErbB2, whereas ErbB4 forms homo- or heterodimers with ErbB2 [27, 28]. The ErbB3 receptor lacks an active kinase domain and is unable to form functional ErbB3 homodimers. The receptor dimerization leads to auto- and trans-phosphorylation of intracellular tyrosine kinase domain with activation of phosphatidylinositol-3-kinase, PI3K/Akt, Ras/extracellular signal-regulated kinase ½, ERK1/2, mitogen-activated protein kinase, MAPK cascade to initiate intracellular signaling events and govern distinct cell-fate decisions [19–23].

NRG1 has been widely studied in stroke [29–33], cardiovascular diseases [34, 35], cerebral malaria [36], and cancer [37, 38]. Studies have demonstrated that NRG1 attenuates tissue damage in acute brain injuries and supports cardiac functions [31–37]. Moreover, exogenous NRG1 treatments alter or inactivate inflammatory pathways associated with tissue damage during ischemic episodes [31–33]. Despite these pieces of evidence, there is a substantial gap in our knowledge about the physiological role of NRG1 in the regulation of cytokines and chemokines in GCs. Therefore, we hypothesize that NRG1-signaling modulates cytokines and chemokines expression and secretion in GCs and ultimately supports fine-tuning the follicular maturation. To address these issues, siRNA-dependent NRG1 was knock-down in gonadotropins primed GCs and evaluated cytokines and chemokines expression and secretions. Subsequently, we determined whether the gain of exogenous-NRG1 impacted the apoptosis induced by a pleiotropic cytokine tumor necrosis factor-α (TNFα) [39]. TNFα is expressed and secreted by GCs and plays a key role in follicular atresia and luteolysis [13, 40–43]. Furthermore, NRG1 and ErbB3 receptors were co-localized in antral-F and POF to demonstrate the gonadotropins-dependent NRG1 and ErbB3 expression pattern in the MGCs, CGCs, and cumulus-oocyte complex (COC).

## Materials and methods

### Animal model and study design

All experiments were approved by the Institutional Animal Care and Use Committee under the guidelines of the National Institutes of Health (NIH) and the US Department of Agriculture. Sprague–Dawley (SD) rats (female, 20–21 days old) were purchased from Charles River Laboratories (USA). Animals were given food and water ad libitum and kept under a regular day/night (12 h light: 12 h darkness) cycle, which was maintained automatically with lighting changes occurring at 0600 and 1800 h. The immature rat has prepubertal ovaries with developing follicles and with undifferentiated GCs. Undifferentiated GCs are unexposed to pubertal cyclic gonadotrophins and lack functional gonadotropin receptors, and do not produce E2 or P4 under basal conditions. Interestingly, undifferentiated GCs respond to gonadotropins with respect to the production of cyclic adenosine monophosphate and induction of gonadotropin-receptor activation of the E2 and P4 biosynthetic pathways. Therefore, to get the homogenous population of GCs from preovulatory follicles, immature SD-female rats (23 days old) were injected subcutaneously (sc) with 10 IU of pregnant mare serum gonadotropin (PMSG, Sigma Aldrich, USA) for 48 h. GCs were isolated from ovaries and cultured as described by Saxena et al. [44].

In brief, ovaries were removed and cleared from the surrounding fat and immediately transferred to a 100-mm cell culture dish containing 15 ml ice-cold Medium 199 (M199; Life Technologies Inc., Invitrogen Corp., Carlsbad, CA, USA) supplemented with 1 mg/ml BSA, 1% Pen-Strep (a mixture of penicillin G and streptomycin, ThermoFisher Scientific, MA, USA). Ovaries were punctured with 25-gauge hypodermic needles and pre-incubated in 6 mM EGTA for 15 min at 37 °C, followed by 5 min incubated at 37 °C in hypertonic sucrose (0.5 M) in M199 medium with 1% Pen-Strep. Thereafter, GCs from each ovary were harvested by manual puncture with 25-gauge needles followed by slight pressure applied with a sterile spatula. Follicular debris was removed manually. Cell clumps and oocytes were removed by filtering the GC suspension through a sterile cell strainer (40-μm nylon mesh, BD Biosciences, USA). The GCs were then pelleted by centrifugation at 1000Xg for 5 min at 4 °C and dispensed into M199 medium supplemented with 10% fetal bovine serum (FBS, GIBCO BRL, Grand Island, NY) and 1% Pen-Strep. Cell viability was evaluated by the trypan blue exclusion technique (https://www.thermofisher.com/us/en/home/references/gibco-cell-culture-basics/cell-cultureprotocols/trypan-blue-exclusion.html). GCs were seeded in six-well plates (~ 10^6^ cells/per well) or 100 mm (∼4–5 × 10^6^ cells/plate) tissue culture plates pre-coated with M199 medium supplemented with 10% FBS and 1% Pen-Strep, and incubated in a humidified atmosphere of 95% O_2_ and 5% CO_2_ at 37 °C. The next morning, medium and unattached cells were removed with fresh media.

After GCs pelleted, a fraction of GCs pellet was washed in M199 medium and frozen at -80 °C until RNA or protein extraction. GCs were also isolated separately from immature female rats (23 days old) ovaries as a control. The purity of differentiated GCs from preovulatory follicles was evaluated for the expression of follicle-stimulating hormone receptor (FSH-R) and aromatase (Cyp19) by Western blot (WB) analysis. GCs from immature rat ovaries were used as a parallel control. The Cyp19 is necessary for the final stages of E2 synthesis and is specifically induced by FSH through FSH-R in the GCs of preovulatory follicles.

Whole ovaries were separately collected from the estrus stage and PMSG-treated rats for immunohistochemistry (IHC).

### Transient transfection of small-interfering NRG1 (siNRG1)

To knock down NRG1 (GenBank: DQ176766.1) expressions, primary rat GCs were transiently transfected with small-interfering-NRG1 (siNRG1) or scramble (negative control with GFP-tag) at 70–80% confluence by using HiPerfect Transfection Reagent (Qiagen, Germantown, MD, USA) according to the manufacturer’s instructions. The siNRG1 was designed at Qiagen using the BIOPREDsi algorithm licensed from Novartis. siNRG1 or scramble RNA transfection was performed in 6-well plates or 100 mm tissue culture dishes. Transfection parameters were optimized according to the instructions provided in the HiPerFect Transfection Reagent handbook. Detail validation of scramble or siRNA can be found on the Web at www.qiagen.com/GeneGlobe. Briefly, scramble or siNRG1 (25 nM based on supplemental results) and HiPerFect were diluted in M199 (Gibco, Grand Island, NY) without serum and incubated for 5 min at room temperature. The cell culture medium was replaced with transfection complexes in a 6-well plate or 100 mm dish and incubated at 37 °C. After 6 h, green fluorescence expression in the scramble-transfected group was checked. Thereafter, cells were maintained in serum-free media for 24, 48, and 72 h in a humidified atmosphere of 95% O_2_ and 5% CO_2_ at 37 °C. The validation of dose and time for siNRG1 were based on supplementary results (Supplementary Fig. 1 given at the end of this article). For validation of siNRG1, three different siNRG1 as siNRG1_a/_b/_c with three different (10, 25, and 50 nM) doses were used for transfection. Post-transfection performance at 24 h was verified by analyzing the percentage inhibition of NRG1 compared with the scramble transfected groups. Effective transfection was defined as resulting in NRG1 knockdown of ~ 70% or greater. Plates displaying less than 60% NRG1 knockdown efficiencies were not analyzed.

The siRNA oligonucleotides target mRNA for degradation. Thus, the degree of NRG1-knockdown was analyzed using Reverse Transcription Polymerase Chain Reaction (RT-PCR) in the scramble and siNRG1 transfected GCs. Moreover, the reduction in transcript expression was analyzed by WB since mRNA levels do not always correlate with protein levels whose protein products have long half-lives. Once knockdown of the NRG1 was confirmed in GCs, its functional effects were further analyzed by evaluating cell survival and apoptosis, expression and secretion of cytokines and chemokines, and phosphorylation of ErbB3/PI3K/AKT/ERK1/2-signaling pathways.

### RNA isolation and purification

RNA was isolated and purified using the RNeasy Mini Kit (Qiagen, Germantown, MD, USA). Cells were lysed in the culture plates directly using a lysis buffer. To the lysate, 70% ethanol was used and passed through the RNeasy spin column to bind RNA. After digesting with DNase to remove any DNA contamination, the column was washed and finally eluted in 30 μl of sRNase-free water (Qiagen, Germantown, MD, USA), according to the manufacturer’s guidelines. The quantity and quality of the isolated RNA from each sample was checked using UV spectroscopy-based analysis. RNA concentration was measured with absorbance (A) at 260 nm (A260), and the purity of RNA content with respect to protein contamination was measured with the ratio A260/A280. All the samples used for the detailed analysis were a 260/280 ratio ~ 2.0 and the 260/230 ratio, which was close to 2.1. The RNA concentrations from different batches varied from 98 to 200 ng/μl.

### Reverse transcription polymerase chain reaction (RT-PCR)

RNA samples were subjected to first-strand cDNA synthesis using iiScript™ cDNA Synthesis Kit (Biorad Laboratories, Hercules, CA, USA). One μg of RNA sample was mixed with 5x iScript reaction mix and iScript reverse transcriptase in a total volume of 20 μl. The complete reaction mixture was incubated in a thermal cycler using the following protocol: priming for 5 mins at 25 °C; reverse transcription for 20 mins at 46 °C; RT inactivation for 1 min at 95 °C.

Gene expression was quantified by qPCR using SsoAdvanced Universal SYBR Green Supermix (Bio-Rad, USA). The cDNA was diluted to 100 μl, and QPCR was done using SsoAdvanced Universal SYBR Green Supermix and CFX connect real-time system (Biorad Laboratories, Hercules, CA, USA). The reaction was set up following the manufacturer’s recommendation in a total volume of 10 μl with 5 μl of supermix, 0.5 μl of each primer, and 2 μl of cDNA. The sequences of the primer set used for the analysis are as follows: Nrg1, 5′-TGAAGGACCTGTCAAACCCG-3′ (forward, F) and 5′-TGCTCCTACTCAGGCAGAGA-3′(reverse, R); ErbB3, 5′-CTGGAATCATGAGGGCGACT-3′(F) and 5′-AGGACACACTGCCTGAGAGT-3′ (R); IL-1a, 5′-ACTCATCGGGAGGAGACGAC-3′(F) and 5′-TCCGGAATCTCCTTCAGCAAC-3′(R); IL-1b, 5′-CAGCTTTCGACAGTGAGGAGA-3′(F) and 5′-TGTCGAGATGCTGCTGTGAG-3′(R); IL-6, 5′-CATTCTGTCTCGAGCCCACC-3′(F) and 5′-GCTGGAAGTCTCTTGCGGAG-3′(R); IL-8, 5′-CCCCCATGGTTCAGAAGATTG-3′(F) and 5′-TTGTCAGAAGCCAGCGTTCAC-3′(R); IL-10, 5′-TGCGACGCTGTCATCGATTT-3′(F) and 5′-TGGCCTTGTAGACACCTTTGT-3′(R); TNFα, 5′-CTCAAGCCCTGGTATGAGCC-3′(F) and 5′-CTCCAAAGTAGACCTGCCCG-3′(R); and GAPDH, 5′-AGGAAATGATGACCTCCTGAACT-3′(F) and 5′-TGTTTTTGTAAGTATCTTGGTGCCT-3′(R). PCRs were performed in a standard 96-well plate format with a Bio-Rad CFX-Connect Real-Time PCR detection system (BioRad i-Cycler, Biorad Laboratories, Hercules, CA, USA). The thermal cycling protocol was programmed as per the manufacturer’s instructions. It was a two-step process: denaturation at 95 °C for 3 min; denaturation at 95 °C for 10 sec followed by the annealing and extension at 55 °C for 30 sec followed by 40 cycles of amplification for all the selected genes. The expressions of each target were measured in triplicate of the eluted RNA. A no-template control (NTC) was analyzed in parallel. To ensure reliable amplification for each individual PCR reaction, PCR melting curve analysis for each target mRNA amplification, which did not meet these criteria, was excluded from further analysis. The raw threshold cycle (*C*_*T*_) value was first normalized to the housekeeping gene (Glyceraldehyde 3-phosphate dehydrogenase, GAPDH) as an internal standard for normalization for each sample to get delta-*C*_*T*_ (Δ*C*_*T*_). The normalized *C*_*T*_ was then calibrated to control cell samples to get delta-delta-*C*_*T*_ (ΔΔ*C*_*T*_) and used to calculate the relative fold expression of the specific gene.

#### Assessment of apoptosis

Apoptosis was measured using standard morphological criteria such as cellular retraction, membrane blebbing, cellular detachment from the plate, and staining [45]. After various treatments, attached cells were collected by trypsinization, and suspended cells were collected by centrifugation. Both the cell fractions were pooled, pelleted, and re-suspended in culture media containing Hoechst33258 (BisBenzimide, Sigma-Aldrich, USA, 1 μg/ml) dye with DAPI (10 μg/ml) at 37 °C for 10–15 minutes. The number of apoptotic cells were quantified on a slide and observed under a microscope. Healthy and apoptotic cells were counted (without sample identity as blinded), and the apoptotic cells were expressed as a percentage of total cells. At least 250–300 cells were counted for each data point.

#### Assessment of chemokines and cytokines in the media 

To determine the effects of NRG1 knock-down on GCs, cytokine and chemokine levels were measured in culture media. The culture media from GCs were collected at 48 hr. post-transfection to analyze cytokines (tumor necrosis factor-α [TNFα], vascular permeability factor/vascular endothelial growth factor [VEGF], interferon-gamma [IFNγ], interleukin IL-1α (IL-1α), IL-2, IL-4, IL-5, IL-6, IL-7, IL-10, IL-12, IL-13, IL-17α), and chemokines (granulocyte-colony stimulating factor [G-CSF], granulocyte-macrophage colony-stimulating factor [GM-CSF], macrophage colony-stimulating factor [M-CSF], monocyte chemoattractant protein-1 (MCP-1/CCL2), macrophage inflammatory proteins 3α [MIP-3α/CCL20], MIP-1β/CCL4, RANTES [CCL5], Keratinocyte chemoattractant (KC)/human growth-regulated oncogene (GRO), erythropoietin (EPO)) using Bio-Plex-Pro-Rat Cytokine, Chemokine, and Growth Factor Magnetic Bead-Based Assays (Bio-Rad, USA) coupled with the Luminex-200 system (Thermofisher, USA). Samples were tested using optimal concentrations of standards and antibodies according to the manufacturer’s protocol. Cytokine, Chemokine, and Growth Factor secretion were expressed in picogram per milliliter (ml) media per milligram (mg) cellular protein (pg/ml/mg).

#### Induction of apoptosis and treatment of recombinant NRG1

The apoptosis was induced in GCs by treating with TNFα (Sigma-Aldrich, USA) in the serum-free medium in the presence or absence of the recombinant-NRG1 (R&D System, USA). The percentage of apoptosis was determined as described above [44]. According to the manufacturer’s guideline, the caspase-3 and -7 activities in living cells were analyzed using the Caspase-Glo-3/7 assay kit (Promega, USA).

#### Immunoblotting

Total protein was extracted after various treatments and subjected to one-dimensional gel electrophoresis and WB analysis [21]. For gel electrophoresis, equal amounts of protein (25μg) were applied to each lane. Primary antibodies were used as described in Table 1. Membranes were incubated with the appropriate secondary antibodies for 2 hr. at room temperature, and antibody binding was detected by chemiluminescence (Pierce, Rockford, IL). Results of representative chemiluminescence experiments were scanned and densitometrically analyzed using a Power Macintosh Computer (G3; Apple Computer, Cupertino, CA) equipped with a Scan Jet 6100C Scanner (Hewlett-Packard, Greeley, CO). The scanned images were quantified using NIH ImageJ version 1.61 software (NIH, Bethesda, MD).

**Table 1:** List of antibodies used for Western blot analysis and immunohistochemistry (IHC)

Peptide/ Protein target	Name of antibody	Name of individual providing the antibody	Species raised (Monoclonal or Polyclonal)	Research Resource Identifier (RRID)	Dilution used
Neuregulin-1β (Nrg1)	Anti-Neuregulin-1β (Nrg1)	Santa Cruz, CA, USA	Rabbit polyclonal	AB_675755	1:1000 (WB) 1:200 (IHC)
ErbB3 receptor	Anti-ErbB3 receptors	Millipore, USA	Mouse monoclonal	AB_309713	1:1000 (WB) 1:200 (IHC)
pErk1/2	Anti-pErk1/2	Cell Signaling, Beverly, MA, USA	Mouse monoclonal	AB_2297442	1:1000
Total Erk1/2	Anti-Total Erk1/2	Cell Signaling, Beverly, MA, USA	Rabbit monoclonal	AB_331775	1:1000
pAkt	Anti-pAkt	Cell Signaling, Beverly, MA, USA	Rabbit polyclonal	AB_329825	1:1000
Total Akt	Anti-Total Akt	Cell Signaling, Beverly, MA, USA	Rabbit polyclonal	AB_329827	1:1000
pPI3K	Anti-pPI3K	Cell Signaling, Beverly, MA, USA	Rabbit polyclonal	AB_659940	1:1000
Total PI3K	Anti-Total PI3K	Cell Signaling, Beverly, MA, USA	Rabbit monoclonal	AB_659889	1:1000
FSH-R	Anti-FSH-R	Abcam, Waltham, MA USA	Sheep polyclonal	AB_2294259	1:1000
Aromatase (Cyp19A1)	Anti- Aromatase (Cyp19A1)	Abcam, Waltham, MA USA	Rabbit monoclonal	AB_444718	1:1000
β-Actin	Anti-Beta-Actin	Cell Signaling, Beverly, MA, USA	Rabbit monoclonal	AB_330288	1:1000

#### Immunohistochemistry (IHC)

To elucidate the expression pattern of NRG1 and ErbB3 in antral and PO-follicles, NRG1 and ErbB3-receptor were co-localized in PMSG treated and estrus phase ovaries as described previously [21, 44]. Negative controls were performed by omitting the primary antibody or using an isotype-matched control antibody derived from the same species. Mounted slides were examined using an Olympus BX41 microscope equipped with an Optronics Magnifier digital camera and Prior Proscan motor-driven stage (Olympus, Melville, NY). To create the final figures, the representative photomicrographs were arranged using Adobe Photoshop (Adobe Systems, San Jose, CA, USA) without any further adjustment to maintain the true nature of the findings.

#### Statistical analysis

All experimental data are expressed as mean ± SEM of three independent experiments (*n* = 3). In each experimental group, GCs cultured from two ovaries/rat/sample. Statistical analysis was carried out by unpaired t-test, one- or two-way ANOVA using SPSS version 11.0. Multiple comparisons were done by Newman-Keuls’ test. Differences were considered to be significant at *P* ≤ 0.05.

## Results

### Knock-down of neuregulin-1 promotes interleukins (IL-1α, IL-1β, IL-6, IL-8, IL-10) and TNFα expression in granulosa cells

To examine whether NRG1 plays a direct role in differentiated GCs, PMSG primed GCs were collected. The purity of GCs was checked by evaluating GCs specific markers, mainly FSH-R and aromatase (Cyp19A1). As shown in Supplemental Fig. 1A, differentiated GCs expressed FSH-R and aromatase, whereas undifferentiated GCs lack these markers at the protein level.

To directly test the potential physiological responses of NRG1 on cytokines and chemokine expression in GCs, the expression of NRG1 was knocked down by transient transfection using siNRG1 in vitro. Studies to determine the efficiency (dose and time-dependent effects) and specificity of siNRG1 knock-down were performed with three different types of siNRG1 namely, siNRG1_a/_b/_c (Supplemental Fig. 1B). Transfection performance was verified by analyzing the degree of silencing obtained with the scramble transfected groups. The siNRG1_c significantly and consistently knock-down NRG1 (65, 80, and 77%) in all the doses (10, 25, and 50 nM at 24 h) in GCs, whereas siNRG1_a/_b was unable to knock-down NRG1 in all the doses. Interestingly, scramble transfected GCs had no adverse effects on NRG1 expression under these experimental conditions. Based on different types of siNRG1 and dose response studies, siNRG1_c was selected as siNRG1 (25 nM dose) for the detailed studies. The functional effects of NRG1 (siNRG1_c) knock-down at 25 nM dose were further analyzed by evaluating cell survival, and apoptosis (Supplemental Fig. 1C) since NRG1 is a pro-survival factor in different cell types including GCs [21–23]. Under NRG1 knock-down condition, GCs survival status was measured in a time course. There was significant apoptotic cell death in siNRG1 (25 nM dose) transfected GCs (10.67 ± 2.08, 20 ± 2.89, and 77 ± 5.56 at 24, 48, and 72 h, respectively) (Supplemental Fig. 1C). In contrast, there was only a small percentage of apoptotic cell death in scramble transfected GCs.

**Fig. 1:**
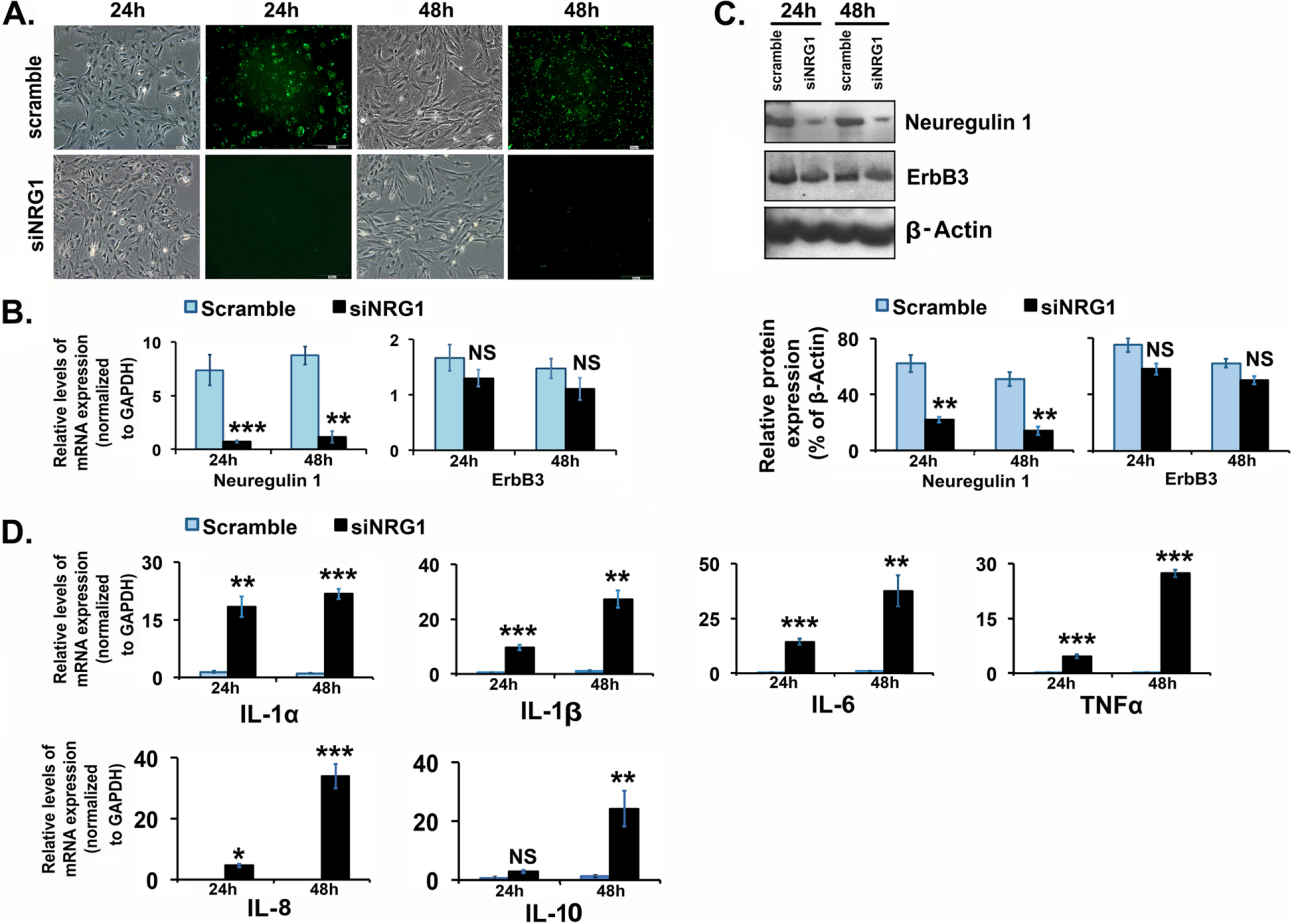
Effects of neuregulin-1 (NRG1) knockdown in granulosa cells (GCs). Pregnant Mare Serum Gonadotropin (PMSG) primed GCs were isolated from two ovaries per rat per sample and grown in culture dishes. GCs were transiently transfected with small-interfering-NRG1 (siNRG1) RNA and scramble-green-fluorescent-protein (GFP) RNA (negative control). Thereafter, cells were maintained in serum-free media and harvested at 24 and 48 h. **A** Representative images of post-transfection live-cell phase (Bright field) and green-fluorescence (scramble) show the transfection efficiency in GCs. **B** and **D** NRG1 and selective cytokines (interleukin (IL)-1α, IL-1β, IL-6, IL-8, IL-10 and tumor necrosis factor-α (TNFα) mRNA expression levels were detected by real-time qPCR in the scramble and siNRG1 transfected group. The levels of mRNA were normalized to Glyceraldehyde 3-phosphate dehydrogenase (GAPDH). **C** Representative immunoblots showing NRG1 and ErbB3 protein expression in GCs. Equal amounts of protein were applied to each lane. *β*-actin was used as an internal control. All bar graphs represent the mean ± *SEM* of results from three individual experiments (*n* = 3). Bar diagrams for protein represent the densitometric analyses of NRG1 and ErbB3 of immunoblots. Asterisks (*) represent unpaired Student t-test, *p ≤ 0.01, **p ≤ 0.001, ***p ≤ 0.0001, NS-not significant

As shown in Fig. 1A, a greater than 70% of GFP-tag scramble transfected GCs in primary culture exhibited the green fluorescent without any adverse effects on cellular morphology. Both scramble and siNRG1 transfected GCs have no morphological difference. Both scramble and siNRG1 transfected GCs have typical epithelial-like morphology with no apparent differences at 24 h and 48 h post-transfection as shown by the phase-contrast photomicrograph and were homogenously transfected with uniform green-immunofluorescence in the scramble group. A time-course study demonstrated that the selected siNRG1 dose significantly down-regulated the expression of NRG1 both at mRNA and protein levels in GCs compared to the scramble group (Fig. 1B and C). Interestingly, the down-regulation of Nrg1 did not affect the expression levels of ErbB3 both at mRNA and protein levels (Fig. 1B and C), which is a bona fide receptor for NRG1.

To define the involvement of NRG1 in the regulation of cytokines and chemokines expression in GCs, a few selected cytokines (IL-1α, IL-1β, IL-6, IL-8, IL-10, and TNFα) expression were analyzed at the mRNA level in NRG1-knockdown GCs post-transfection. We found a significant higher of expression of IL-1α, IL-1β, IL-6, IL-8 and TNFα in siNRG1-transfected GCs compared to scramble transfected GCs at 24 h and 48 h, except IL-10 which was higher level at 48 h (Fig. 1D). Interestingly, IL-1α, IL-1β, IL-6, IL-8, IL-10, and TNFα are 1.5, 1.6, 1.2, 3.7, 4.1 and 6.1 respectively, a higher fold of expression at 48 h compared to those at 24 h in the siNRG1-transfected GCs.

### Knock-down of neuregulin-1 promotes differential cytokines and chemokines secretion in granulosa cells

**Fig. 2:**
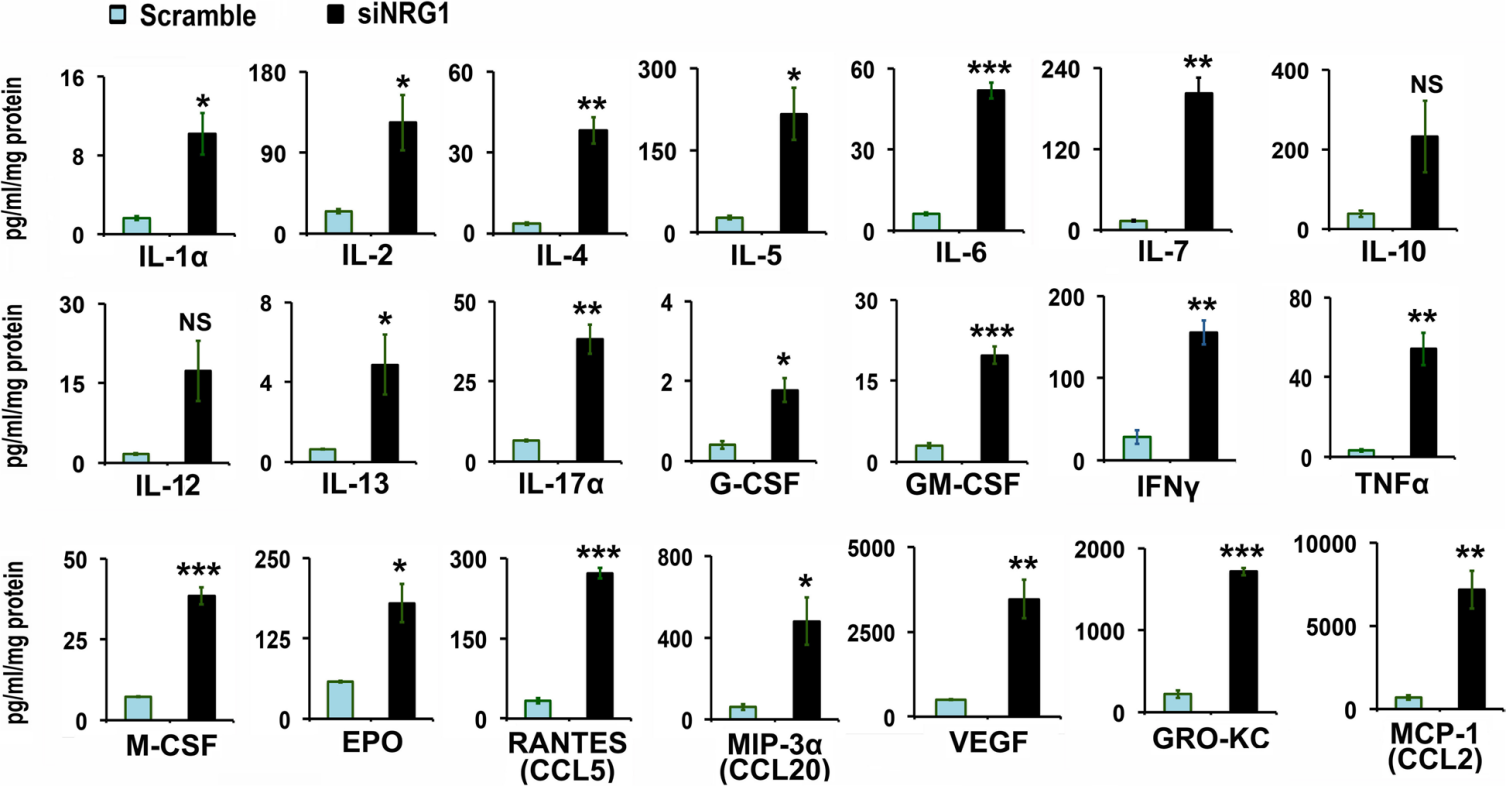
Effects of neuregulin-1 (NRG1) knockdown on chemokines and cytokines secretion in granulosa cells (GCs). Pregnant Mare Serum Gonadotropin (PMSG) primed GCs were isolated from two ovaries per rat per sample and grown in culture dishes. GCs were transiently transfected with small-interfering-NRG1 (siNRG1) RNA and scramble-green-fluorescent-protein (GFP) RNA (negative control). Thereafter, cells were maintained in serum-free media, and media were collected at 48 h. Concentrations of chemokines and cytokines were measured and analyzed in media using Bio-Plex-Pro-Rat Cytokine, Chemokine, and Growth Factor Magnetic Bead-Based Assays (Bio-Rad, USA) coupled with the Luminex-200 system. Cytokine, Chemokine, and Growth Factors are expressed in picogram per ml per milligram (pg/ml/mg) cellular protein. All bar graphs represent the mean ± *SEM* of results from three individual experiments (*n* = 3). Asterisks (*) represent unpaired Student t-test, *p ≤ 0.01, **p ≤ 0.001, ***p ≤ 0.0001, NS-not significant. Abbreviations- IL: Interleukin, G-CSF: granulocyte colony-stimulating factor, GM-CSF: granulocyte-macrophage colony-stimulating factor, GRO-KC: Keratinocyte chemoattractant (KC)/human growth-regulated oncogene (GRO), IFNγ: Interferon-gamma, MCSF: Macrophage-colony stimulating factor, MCP1: Monocyte chemoattractant protein-1, MIP-3α: Macrophage Inflammatory Protein 3α, TNFα: tumor necrosis factor α, VEGF: vascular permeability factor/vascular endothelial growth factor, RANTES: Regulated upon Activation, Normal T Cell Expressed and Presumably Secreted.

To better understand the cytokines and chemokines profile of NRG1 knock-down GCs, protein levels of cytokines and chemokines were analyzed by Cytokine, Chemokine, and Growth Factor Assay in the culture media. Based on higher IL-1α, IL-1β, IL-6, IL-8, IL-10, and TNFα mRNA expression level in NRG1 knock-down GCs, the cytokines and chemokines secretions were measured in the media at 48 h. We observed under basal condition a low-level of various cytokines and chemokines are secreted differentially in the scramble transfected GCs (Fig. 2). Interestingly, similar to IL-1α, IL-1β, IL-6, IL-10, and TNFα mRNA expression in NRG1 knock-down GCs, the cytokines and chemokine secretion at protein levels were dramatically and significantly (*p* > 0.05, and Student’s *t*-test) increased in the secretions of NRG1 knock-down GCs (Fig. 2). The mean range of secretion of cytokines and chemokines were 3–70 pg/ml/mg protein (IL-1α, − 4, − 6, − 13, − 17α, G-CSF, GM-CSF, TNFα and M-CSF), 120–660 pg/ml/mg protein (IL-2, − 5, − 7, IFNy, EPO, RANTES and MIP-3α), and 3500–8000 pg/ml/mg protein (VEGF, GRO-KC and MCP-1) in NRG1 knock-down GCs. Moreover, few cytokines and chemokines proteins, namely, IL-1β and IL-18, were at or below the limit of detectability in media from both siNRG1 and scramble transfected GCs. Moreover, there is a significant higher mean fold change in the secretion for the cytokines and chemokine (IL-1, − 2, − 4, − 5, − 6, − 7, − 13, − 17, G-CSF, GM-CSF, TNFα, M-CSF, IFN-γ, EPO, RANTES, MIP-3α, VEGF, GRO-KC and MCP-1 ranging 6.3, 5.0, 10.3, 8.2, 8.3, 15.2, 7.6, 5.8, 4.3, 6.6, 16.6, 5.3, 5.4, 3.1, 8.1, 7.9, 7.0, 7.7, and 10.3 respectively) in NRG1 knock-down GCs compared to scramble transfected GCs at 48 h, except IL-10 and IL-12 (not significant). Off note, IL-8 was not available in rat Bio-Plex-Pro-Rat Cytokine, Chemokine, and Growth Factor Magnetic Bead-Based Assays (Bio-Rad, USA). Taken together, these results suggest that the cytokines and chemokines were secreted differentially but significantly at higher magnitude and concentrations in the media of the siNRG1-transfected GCs compared to the scramble group.

### Knock-down of neuregulin-1 inhibits phosphorylation of PI3K/Akt and ERK1/2 in granulosa cells

**Fig. 3:**
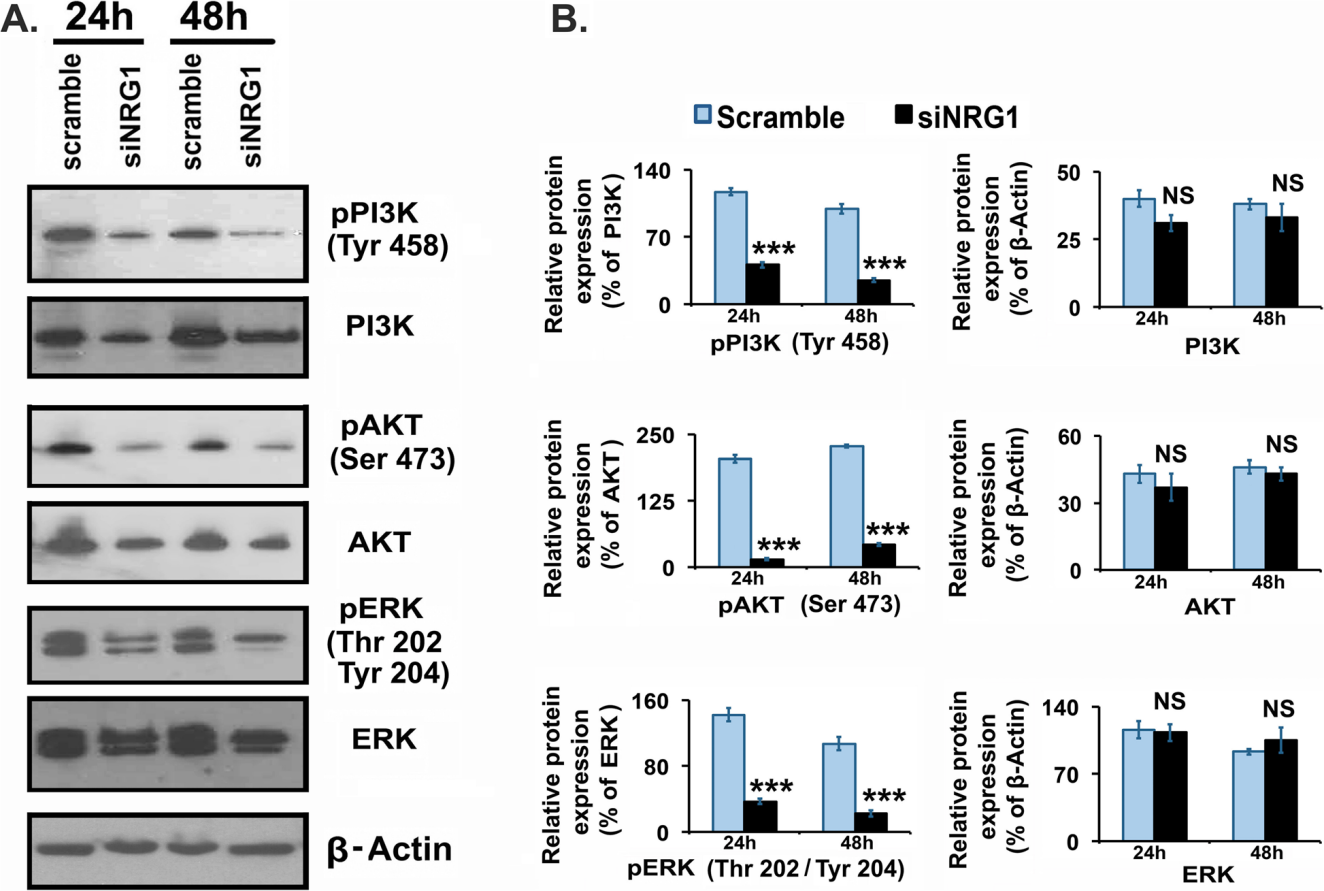
Effects of neuregulin-1 (NRG1) knockdown on kinases in granulosa cells (GCs). Pregnant Mare Serum Gonadotropin (PMSG) primed GCs were isolated from two ovaries per rat per sample and grown in culture dishes. GCs were transiently transfected with small-interfering-NRG1 (siNRG1) RNA and scramble-green-fluorescent-protein (GFP) RNA (negative control). Thereafter, cells were maintained in serum-free media and harvested at 24 and 48 h. Representative immunoblots showing phospho(p)- and total phosphoinositide 3-kinase (PI3K), serine/threonine-protein kinase (AKT/Protein Kinase B) and extracellular signal-regulated kinases (ERK1/2) protein expressions in GCs. Equal amounts of protein were applied to each lane. *β*-actin was used as an internal control. Bar diagrams represent the densitometric analyses of immunoblots. All bar graphs represent the mean ± *SEM* of results from three individual experiments (*n* = 3). Asterisks (*) represent unpaired Student t-test, *p ≤ 0.01, **p ≤ 0.001, ***p ≤ 0.0001, NS-not significant

Studies have shown that NRG1 through ErbB-receptors stimulate major downstream signaling targets are PI3K/Akt and Erk1/2, MAPK in different cell types [19–23, 26–28]. Therefore, the total and the phosphorylation of PI3K/Akt and Erk1/2 were analyzed to clarify whether PI3K/Akt and Erk1/2 signaling pathways were affected in response to NRG1 knock-down in GCs. Under siNRG1-dependent knock-down of NRG1 significantly (*P* < 0.001) reduced the phosphorylation of both PI3K/Akt and ERK1/2 in GCs (Fig. 3). In contrast, the phosphorylation of PI3K/Akt and Erk1/2 were maintained in scramble transfected GCs (Fig. 3). These experimental results suggest that depletion of NRG1 had a negative impact on phosphorylation of PI3K/Akt and Erk1/2 without changing the total PI3K/Akt and Erk1/2 expression levels in GCs.

### Exogenous recombinant Neuregulin-1 treatment attenuates the effects of TNFα in serum-starved granulosa cells

**Fig. 4:**
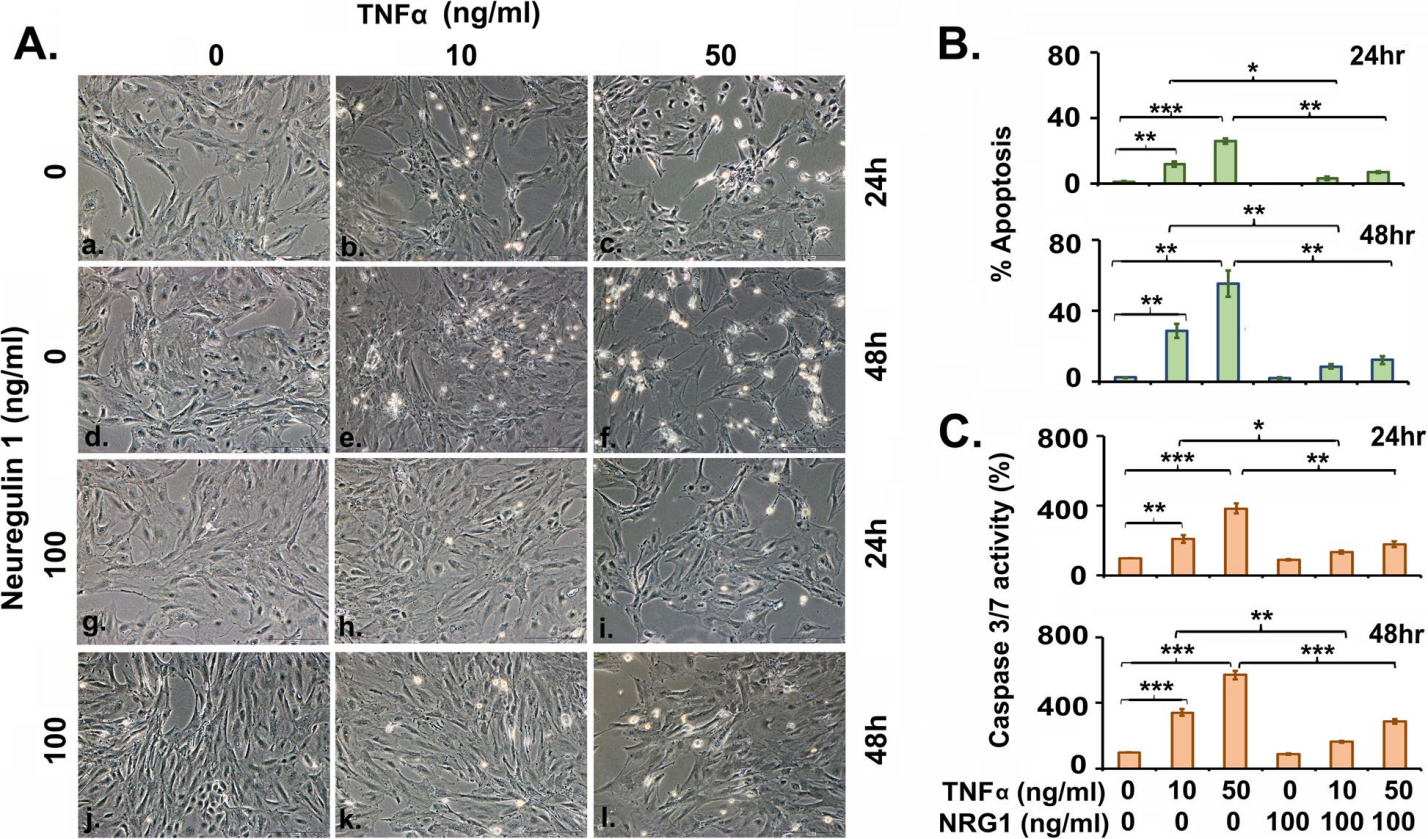
Exogenous neuregulin-1 effects on tumor necrosis factor α (TNFα) induced apoptosis in granulosa cells. Pregnant Mare Serum Gonadotropin (PMSG) primed GCs were isolated from two ovaries per rat per sample and grown in culture dishes. Thereafter, GCs were treated with TNFα (10 and 50 ng/ml) in the presence or absence of recombinant-NRG1 (100 ng/ml) in serum free media. **A** Representative live-cell images were taken under an inverted microscope at X200 magnification at 24 and 48 h post-treatment to represent the morphological changes of cells. **B** The bar graph represents the percentage of cells displaying nuclear morphologic changes during apoptosis. **C** Caspase-3/7 activity in GCs was measured as described in Material and methods. All bar graphs represent the mean ± *SEM* of three individual experiments. Asterisks (*) represent unpaired Student t-test, *p ≤ 0.01, **p ≤ 0.001, ***p ≤ 0.0001

To investigate the effects of NRG1-signaling in the readily tractable model, we treated serum-starved GCs culture with or without NRG1 in the presence of exogenous cytokine TNFα (Fig. 4A-C). In GCs, TNFα potently and significantly caused apoptotic cell death in a dose and time-dependent manner (Fig. 4A-B). To determine the mechanism by which cell death induced by TNFα in GCs, the levels of the effector caspases-3 and -7 activities were measured. Caspase-3 and caspase-7 are activated universally during apoptosis, irrespective of the specific death-initiating stimulus. Both proteases are widely considered to coordinate the demolition phase of apoptosis by cleaving a diverse array of protein substrates [46]. Consistent with the hypothesis that NRG1 protects GC, co-treatment of GCs with exogenous NRG1 profoundly repressed the exogenous TNFα-dependent activation of the caspase-3/7 activity and maintained GCs survival. In contrast, in the absence of exogenous-NRG1, caspase-3/7 activity was significantly very high (2–3 fold at 24 h and 3–6 fold at 48 h over the control group) with higher cell death in the presence of exogenous cytokine TNFα treated GCs (Fig. 4A-C). These results highlight a major role of NRG1 in the survival of GCs exposed to TNFα in vitro.

### Gonadotropin dependent expression of NRG1 and ErbB3 in antral and preovulatory follicle

**Fig. 5:**
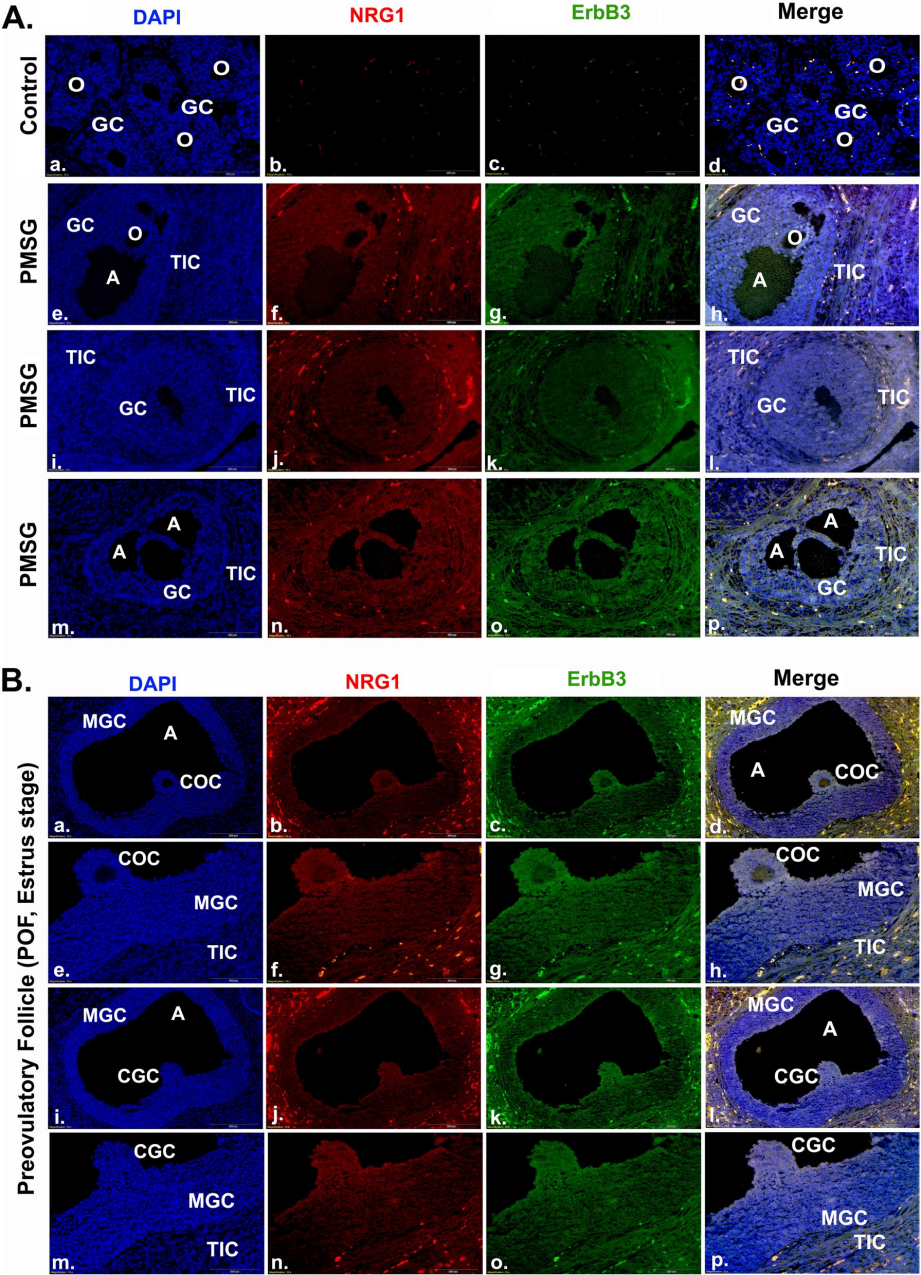
Representative images of immunocolocalization of neuregulin-1 (NRG1) and epidermal growth factor receptor 3 (ErbB3) in the rat ovarian follicles. Ovaries were collected from pregnant mare serum gonadotropin (PMSG)-treated and the estrus stage rats for immunohistochemistry (IHC) (*n* = 3 rats per group). Immunolocalization of endogenous NRG1 and ErbB3 were with Alexa Fluor 594-labeled (red) and Alexa Fluor 488-labeled (green) secondary antibodies respectively. The nucleus was counter stained with 4′,6′-diamidino-2-phenylindole (blue). **A** Representative photomicrographs of transverse- and sagittal-sections of antral-follicle in PMSG-treated ovary. **B** Transverse section of pre-ovulatory follicle (POF) during estrus stage showing cumulus-oocyte-complex (COC). A-antrum, GC-granulosa cells, C-cumulus, M-mural, O-oocyte, TIC-theca-interstitial cells

NRG1 and ErbB3 were co-localized in GCs during antral and PO follicular development and differentiation to reconcile the effects of both exogenous (PMSG) and endogenous gonadotrophins on NRG1 and ErbB3-expression. PMSG treatment was used as the source of exogenous gonadotrophin to promote follicular maturation in immature rats, whereas cyclic rat ovaries from the normal estrus phase were used to show the direct co-relative patterns of NRG1 and ErbB3-expression in GCs in normal follicular maturation vs. forced maturation of follicle. PMSG treatment promotes immature follicle to the differentiated follicle. The transverse- and sagittal sections of antral-follicles in PMSG-stimulated ovaries showed an intensely co-localized NRG1 and ErbB3 in GCs (Fig. 5A). Similarly, NRG1 and ErbB3 were intensely co-localized in the MGCs, CGCs, and COC of POF in the estrus stage as a “circular ring of protein” around the ova (Fig. 5B). The theca interstitial cells also showed intense immunostaining for NRG1 and ErbB3 in both PMSG primed antral follicle and POF from cyclic rat ovaries. However, no detectable immunostaining was observed when the primary antibodies were replaced with IgG, confirming the specificity of the staining procedures. These results suggest a possible NRG1 and ErbB3 receptor role in both antral- and PO-follicle.

## Discussion

In the current study, we have demonstrated a new role for NRG1-signaling in regulating cytokines and chemokine expression and secretion in GCs and supporting the pro-survival of GCs. The dynamic nature of follicular development and oocytes in the follicular fluid surrounded by the MGCs and CGCs make the follicle a unique structure [1]. Moreover, GCs prevent oocyte abnormal nuclear and cytoplasmic maturation [47, 48]. The current findings corroborated with previously published data that immune signaling molecules, namely cytokines and chemokines, are differentially expressed and secreted at a low level by the GCs in the basal condition to support normal follicular maturation [2–5, 10, 11, 14]. The gonadotropins-dependent optimal levels of cytokines and chemokines, their complicated signaling network, and the direction of the response are the hallmarks of many processes in reproductive physiology. These include antral follicle and POF formation and maturation, ovulation, CL formation, luteolysis, menstruation, and implantation [2–5, 10, 11, 13, 14, 40, 43, 49]. Additionally, we have demonstrated that inhibition of NRG1 expression in GCs promotes uncontrolled cytokines and chemokines expression and secretion [29–38]. A differential and distinct magnitude and concentrations of cytokine and chemokine expression and secretion patterns in NRG1 knock-down GCs suggest that cytokine and chemokine expression and secretions may be governed partly by the NRG1 expression in the GCs.

Interestingly, the activation of pro-inflammatory and pro-apoptotic cytokines and chemokines are also accompanied by the simultaneous increase in the synthesis and secretion of their antagonist cytokines and chemokines, including IL-4, − 10, − 12, GM-CSF, and VEGF, which promote the anti-inflammation and pro-survival of cells by inhibiting a local inflammatory response [2, 10, 13, 40, 43, 50–65]. These compensatory anti-inflammatories and pro-survival effects may be small to prevent the imbalance of high magnitude of the amplification of pro-inflammatory and pro-apoptotic cytokines and chemokines effects in current experimental conditions through unknown mechanisms. To regulate follicular development, maturation, and atresia, a wide range of crucial negative and positive feedback signaling loops have evolved in nature. In this regard, NRG1 may have emerged as a critical molecular regulator to control cytokines, chemokines, and fine-tune signaling to properly resolve inflammation and prevent uncontrolled follicular maturation or atresia. The uncontrolled cytokines and chemokines can modulate the translation of transcripts resulting in increased levels of immunomodulating factors that can promote the inflammatory response. Thus, NRG1 may act as an important regulator to balance cytokines and chemokines expression and secretion [2, 10, 13, 40, 43, 50–65]. It has demonstrated that abnormal acceleration of reproductive aging occurs in GC-specific Nrg1 knockout mice due to the endocrine and matrix alteration within the ovarian stroma [64]. In contrast, uncontrolled or abundance of intra-ovarian cytokines and chemokines expression adversely affect ovarian reserve with pathophysiological aging [66–69]. Acute and chronic ovarian and uterine inflammation can negatively affect hormone production, ovulation, and fertility [9, 43, 70]. IL-1α, IL-1β, ILs-6, and TNFα are among the cytokines that are most commonly found to increase at the intra-organ and systemic levels during inflammaging [70]. One cause of decreased fertility during the post-partum period with a prolonged anovulatory status is a higher concentration of inflammatory cytokines [14, 70]. Thus, these results may have clinical implications.

Present results demonstrate a direct effect of NRG1 knock-down on phosphorylation of PI3K/AKT/ERK1/2 [21–23, 71–75]. These observations suggest that possibly ErbB3-phosphorylation is affected due to the knock-down of NRG1-ligand in GCs and interrupted the phosphorylation of PI3K/AKT/ERK1/2 [21–23, 71–75]. NRG1-dependent activation of PI3K/AKT/ERK1/2-signaling through ErbB3 is a potential key signaling pathway in different cell types demonstrated mainly through exogenous NRG1 treatment, including our previous studies [19–23, 26–28, 71–75]. ErbB3 loss in the mammary epithelium of mice impaired Akt and MAPK signaling and reduced luminal cell survival and proliferation with increased expression of multiple cytokines [71–75]. Thus, NRG1 knock-down may be a consequence of altered pPI3K/pAkt/pERK1/2-phosphorylation and cytokines and chemokines expression and secretion in GCs. These inverse correlations between pPI3K/pAkt/pERK1/2 and cytokines and chemokines expression and secretion suggest a possible regulatory link through NRG1 in the GCs.

Our studies further suggest that exogenous NRG1 treatment prevents TNFα-induced cell death and supports the pro-survival of GCs. TNFα coordinates tissue homeostasis by regulating cell survival and death by activating downstream effector caspases-3/7 [39, 46, 76–78]. The proteolytic maturation and activation of caspase-7 is dependent on inflammasome (caspase-1 complex) and has been observed under inflammatory conditions [46, 79, 80]. Similarly, caspase-3 activation is the caspase-8 and -9 protein complex dependent during apoptosis [79, 80]. Our experimental results suggest that exogenous NRG1 prevents pro-inflammatory cytokine TNFα-dependent GC death and promotes cell survival [19–23, 79, 80]. Moreover, the co-localization of NRG1-ErbB3 in the GC, CGC, and MGC of antral and PO-follicles suggest that gonadotropins-dependent NRG1 expressions may act as a ligand for ErbB3 in GC, MGC, and CGC of antral and PO-follicle [21–23, 81].

Together, current in vitro results and co-localization of NRG1-ErbB3 in antral and PO follicle suggest that NRG1-ErbB3 signaling in GCs provide an extra layer in orchestrating the follicular microenvironment for oocyte competency during the advanced antral- and PO follicular stage [2–7, 20–23, 81–84].

In conclusion, the present findings provide new information on NRG1-signaling in GCs that may involve balancing and coordinating somatic cell-oocyte interactions through cytokines and chemokines expression and secretion. Ultimately, NRG1-signaling in GCs fine-tunes the follicular maturation, ovulation, and prevention of atresia. However, further studies are needed using genetic gain or loss-of-function models of individual selected cytokines and chemokine to pinpoint the biological effects of NRG1-signaling that influence the GCs fate decisions within the antral follicle and POF to shape the follicular maturation, oocyte competence for ovulation and prevents follicular atresia.

## Supplementary Information

**Additional file 1: Supplemental Fig. 1. A.** Pregnant Mare Serum Gonadotropin (PMSG) primed, and immature rat ovarian granulosa cells (GCs) were isolated. Total protein of undifferentiated and differentiated GCs was extracted and analyzed for follicle-stimulating hormone receptor (FSH-R) and aromatase (Cyp19A1) using Western blots. Equal amounts of protein were applied to each lane. *β*-actin was used as an internal control. **B.** To elucidate the physiological and functional responses of neuregulin 1 (NRG1) in GCs, we performed knock-down studies of NRG1 expression in GCs using siNRG1. Studies to determine the efficiency and specificity of siNRG1-dependent knock-down of NRG1 expression were performed with three different siNRG1 as siNRG1_a/_b/_c with three different (10, 25, and 50 nM) doses for transfection. mRNA expression levels were detected post-transfection by real-time qPCR in the scramble and siNRG1 transfected group at 24 h. The levels of mRNA were normalized to Glyceraldehyde 3-phosphate dehydrogenase (GAPDH). **C.** Pregnant Mare Serum Gonadotropin (PMSG) primed GCs were isolated from two ovaries per rat per sample and grown in culture dishes. GCs were transiently transfected with small-interfering-NRG1 (siNRG1_c) and scramble RNA (negative control) at 25 nM concentration. After various treatments, the number of apoptotic cells was quantified. The apoptotic cells were expressed as a percentage of total cells at 24, 48, and 72 h. For detail, see Materials and methods. All bar graphs represent the mean ± *SEM* of three individual experiments. Asterisks (*) represent unpaired Student t-test, *p ≤ 0.01, **p ≤ 0.001, ***p ≤ 0.0001, NS-not significant.
