# Supplementary figures and images for: Neuregulin-1 signaling regulates cytokines and chemokines expression and secretion in granulosa cell
Source: J Ovarian Res. 2022 Jul 26;15:86. doi: 10.1186/s13048-022-01021-0 (PMC9316729; doi:10.1186/s13048-022-01021-0)

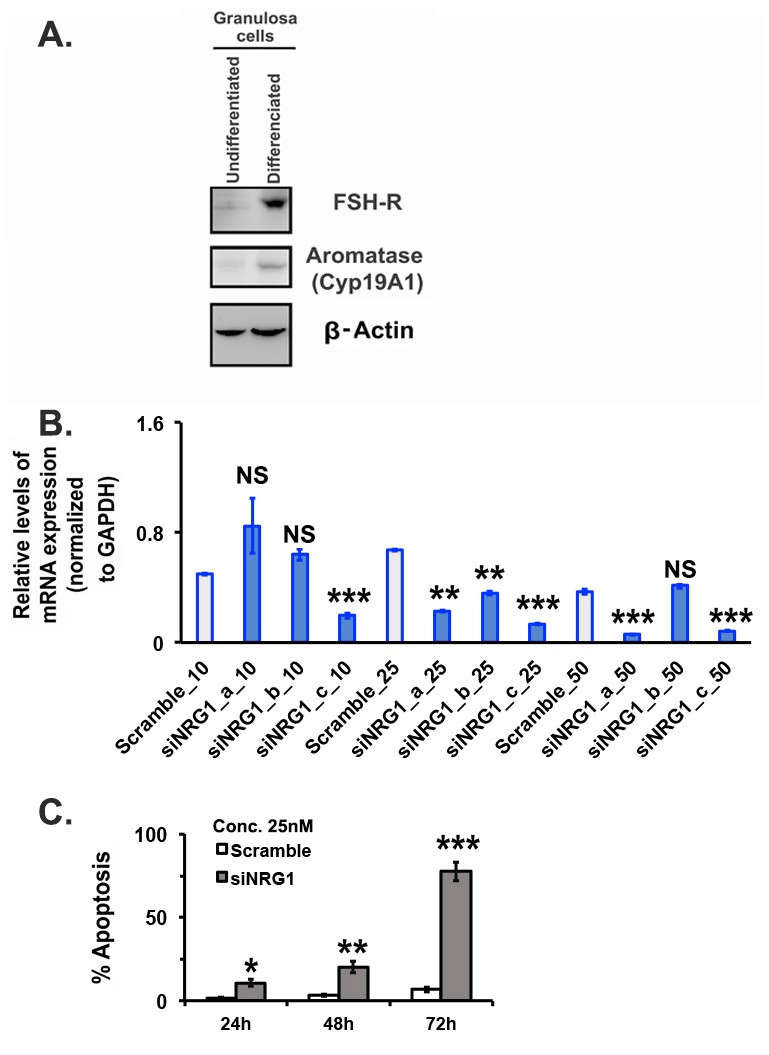

Supplement: Supplementary file 1 — Additional file 1: Supplemental Fig. 1. A. Pregnant Mare Serum Gonadotropin (PMSG) primed, and immature rat ovarian granulosa cells (GCs) were isolated. Total protein of undifferentiated and differentiated GCs was extracted and analyzed for follicle-stimulating hormone receptor (FSH-R) and aromatase (Cyp19A1) using Western blots. Equal amounts of protein were applied to each lane. β-actin was used as an internal control. B. To elucidate the physiological and functional responses of neuregulin 1 (NRG1) in GCs, we performed knock-down studies of NRG1 expression in GCs using siNRG1. Studies to determine the efficiency and specificity of siNRG1-dependent knock-down of NRG1 expression were performed with three different siNRG1 as siNRG1_a/_b/_c with three different (10, 25, and 50 nM) doses for transfection. mRNA expression levels were detected post-transfection by real-time qPCR in the scramble and siNRG1 transfected group at 24 h. The levels of mRNA were normalized to Glyceraldehyde 3-phosphate dehydrogenase (GAPDH). C. Pregnant Mare Serum Gonadotropin (PMSG) primed GCs were isolated from two ovaries per rat per sample and grown in culture dishes. GCs were transiently transfected with small-interfering-NRG1 (siNRG1_c) and scramble RNA (negative control) at 25 nM concentration. After various treatments, the number of apoptotic cells was quantified. The apoptotic cells were expressed as a percentage of total cells at 24, 48, and 72 h. For detail, see Materials and methods. All bar graphs represent the mean ± SEM of three individual experiments. Asterisks (*) represent unpaired Student t-test, *p ≤ 0.01, **p ≤ 0.001, ***p ≤ 0.0001, NS-not significant. [file 13048_2022_1021_MOESM1_ESM.jpg]
